# The versatile multi-functional substance NMN: its unique characteristics, metabolic properties, pharmacodynamic effects, clinical trials, and diverse applications

**DOI:** 10.3389/fphar.2024.1436597

**Published:** 2024-10-01

**Authors:** Bin Yu, Xiaotong Jing, Lina Jia, Maoru Wang, Liying Liu, Songyuge Ping, Yu Wang, Min Yang

**Affiliations:** ^1^ Department of Pharmacy, Mianyang Central Hospital, School of Medicine, University of Electronic Science and Technology of China, Mianyang, China; ^2^ School of Pharmacy, Collaborative Innovation Center of Advanced Drug Delivery System and Biotech Drugs in Universities of Shandong, Key Laboratory of Molecular Pharmacology and Drug Evaluation, Yantai University, Yantai, China; ^3^ Wuhan University School of Nursing, Wuhan University, Wuhan, China; ^4^ Department of Central Sterile Supply Department, Mianyang Central Hospital, School of Medicine, University of Electronic Science and Technology of China, Mianyang, China; ^5^ Drug Dispensing Department, The Third Hospital of Mianyang, Sichuan Mental Health Center, Mianyang, China

**Keywords:** Chinese medicine, food, human clinical trials, medicine, NMN, pharmacologic action

## Abstract

β-nicotinamide mononucleotide (NMN) is a naturally occurring biologically active nucleotide widely present in organisms and an inherent substance in the human body. As a critical intermediate in synthesizing coenzyme I (NAD+), it widely participates in multiple biochemical reactions in the human body and is closely related to immunity, metabolism, and other factors. In recent years, NMN has rapidly developed and made significant progress in medicine, food, and healthcare. However, there is currently a lack of comprehensive reports on the research progress of NMN, as well as exploration and analysis of the current research achievements and progress of NMN. Therefore, this review is based on retrieving relevant research on NMN from multiple databases at home and abroad, with the retrieval time from database establishment to 20 May 2024. Subsequently, literature search, reading, key information extraction, organization, and summarization were conducted with the aim of providing a comprehensive and in-depth analysis of the characteristics, metabolic pathways, pharmacological effects, progress in human clinical trials, and wide applications of NMN in drug development and food applications. Furthermore, it offers personal insights into NMN’s potential future developments and advancements to present the current development state and existing challenges comprehensively. Ultimately, this review aims to provide guidance and serve as a reference for the future application, innovation, and progression of NMN research.

## 1 Introduction

Surgery, chemotherapy and radiotherapy constitute the main treatment methods against tumors (Akbari et al., 2020; Zhang et al., 2023). However, radiotherapy and chemotherapy may also bring some side effects during the treatment. Chemotherapy may affect multiple organ systems in the human body, such as digestive, blood, immune and respiratory systems (Rohrhoff et al., 2014). Since chemotherapy drugs are cytotoxic, they can damage normal cells in addition to cancer cells, which can result in undesirable adverse reactions. In addition, the radiation generated during radiotherapy may also damage normal tissues, exacerbating the risk of side effects. Common adverse reactions of radiotherapy include skin damage, nausea and vomiting, fatigue, bone marrow suppression, gastrointestinal reactions, etc (Klausner et al., 2021).

β-Nicotinamide mononucleotide (β-NMN, the following will unify the β-NMN as NMN) is a natural active nucleotide. As a critical precursor for the production of coenzyme I (Nicotinamide adenine dinucleotide, NAD+), it is ubiquitous in various organisms (Jiang et al., 2022). Recently, researchers have revealed the potential of NMN in alleviating the side effects of radiotherapy and chemotherapy. It can promote the energy metabolism process, strengthen the self-repair and renewal function of cells, and further reduce the damage of radiotherapy and chemotherapy to normal cells by improving the content of NAD+ in cells (Sun et al., 2019; Zhao et al., 2022). Besides, NMN also has antioxidant and anti-inflammatory functions, which can help relieve oxidative stress and inflammatory responses triggered by radiotherapy and chemotherapy and further reduce the severity of side effects (Duan et al., 2023; Song et al., 2023). However, although NMN has many effects, there is no systematic literature to report and analyze it. Hence, this article conducts a comprehensive search across multiple databases to gather relevant research reports on NMN, encompassing various fields such as pharmacokinetics, pharmacodynamics, pharmacology, clinical trials, and the intersecting realms of drugs and food. The core information from this literature is distilled to elucidate NMN’s characteristics, metabolism, pharmacological effects, applications in food and drug industries, and its progression in human clinical trials. The objective is to foster a deeper understanding of the research advancements in diverse NMN fields and present some personal perspectives on its future potential.

## 2 Methods

In order to comprehensively and systematically evaluate and review the unique characteristics, metabolic properties, pharmacological effects, human clinical trials, and various applications of NMN, this study adopted a rigorous and systematic literature search, query, and data extraction strategy. The specific method is as follows: Firstly, determine the search keywords: Based on the theme of this study, core search keywords including “β-nicotinamide mononucleotide”, “nicotinamide mononucleotide”, “NMN”, “NAD+“, “pharmacodynamics”, “pharmacology”, “metabolic characteristics”, “Chinses medicine”, “food” and “human clinical trials”. Secondly, selecting databases and resources: This study selected multiple authoritative academic databases and literature search platforms at home and abroad, such as Web of Science, PubMed, Embase, Clinical Trials Website, China National Knowledge Infrastructure (CNKI), Chinses Wanfang and Chinses VIP databases, etc. The language of the literature is English or Chinese. The retrieval covers the period from the establishment of the database to 20 May 2024. In addition, the types of literature include reviews, meta-analysis, original articles, conference papers, patents, dissertations, etc. Subsequently, text reading and data extraction: Read the titles and abstracts of the preliminarily screened literature to further confirm whether they fulfill the inclusion criteria. During the confirmation process, detailed information such as the author, publication year, research purpose, methods, results, and conclusions of each literature were recorded. Finally, data integration and analysis: After completing data extraction, integrate and analyze the collected information. Based on the different characteristics and application fields of NMN, classify and summarize relevant information to form a systematic review framework. Meanwhile, conduct a comprehensive analysis of key research findings. Furthermore, we have repeatedly verified and revised the preliminary conclusions to ensure the accuracy and comprehensiveness of the review content.

This study aims to comprehensively and objectively evaluate and summarize the unique characteristics, metabolic properties, pharmacological effects, human clinical research, and diverse applications of NMN. Through the literature search, query, and data extraction methods outlined in the above system, it provides valuable reference information for researchers and clinical practitioners in related fields.

## 3 Overview of NMN

### 3.1 Physicochemical properties of NMN

**FIGURE 1:**
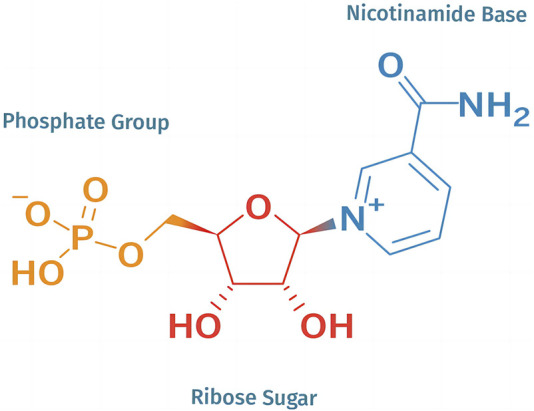
Molecular structure of NMN.

NMN is one of the isomers of nicotinamide mononucleotide. Nicotinamide mononucleotide includes two isomers, α and β, but only β is an active isomer, as shown in Figure 1. The chemical molecular formula of NMN is C_11_H_15_N_2_O_8_P, and the molecular structure consists of three parts: nicotinamide base, ribose sugar and a phosphate group (Ou et al., 2024). In appearance, NMN is a crystalline powder from white to slightly yellow, with no significant odour, and should be stored in a dry environment at a temperature of −20°C under dark conditions. It has a melting point of about 166°C and a boiling point of °C at a pressure of 760 mmHg. NMN can be rapidly dissolved in water, but its solubility in acetone is extremely low. Its pH value is usually between 3.0 and 4.0. As a natural active nucleotide, NMN is an inherent substance in the human body and is widely distributed in certain fruits and vegetables (Alegre and Pastore, 2023).

### 3.2 Metabolic characteristics of NMN

**FIGURE 2:**
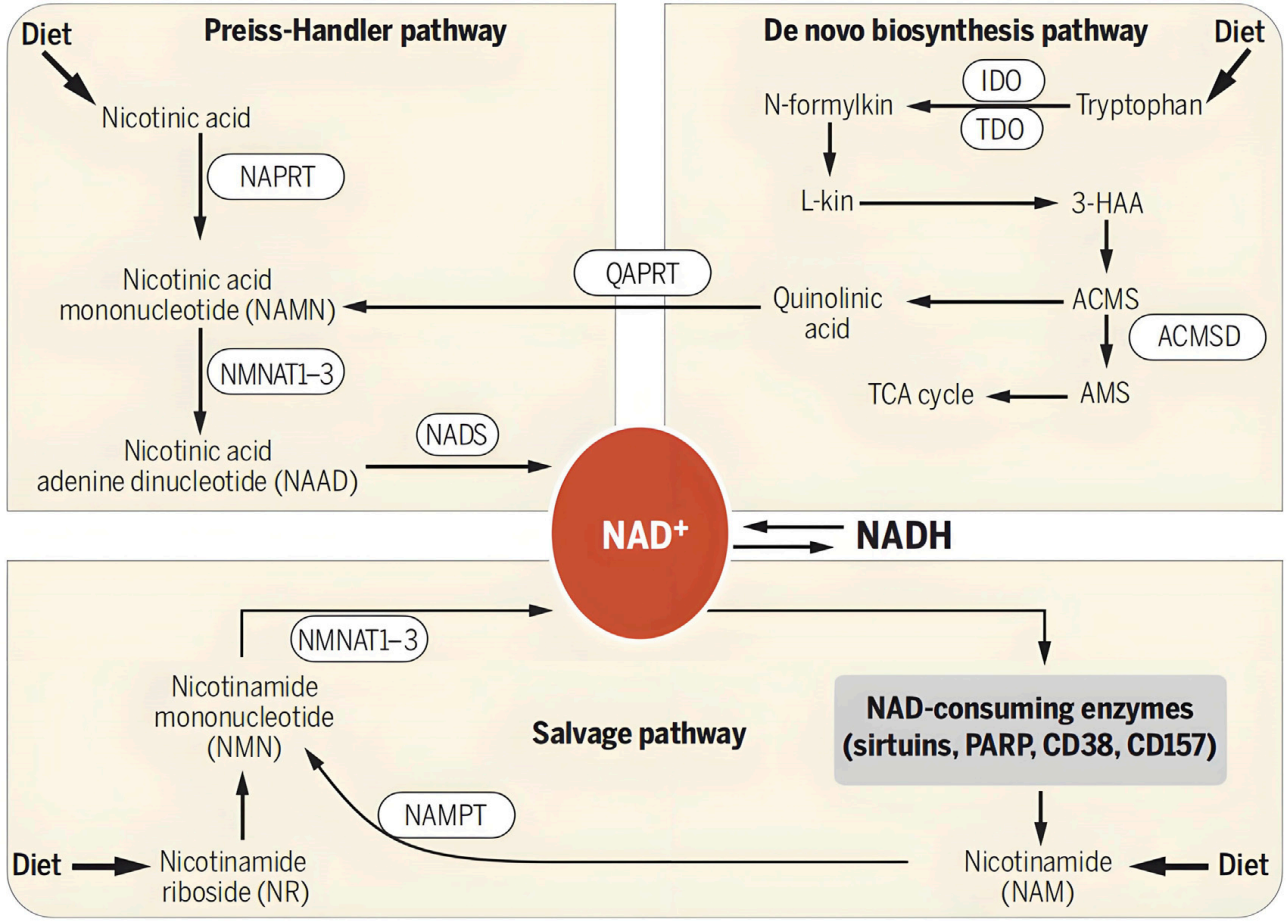
Formation and metabolism of NMN in human body. NAD + levels are maintained by three independent pathways. First, the Preiss-Handler pathway uses dietary nicotinic acid and the enzyme nicotinic acid phospho ribosyltransferase (NAPRT) to generate NAMN, which is then transformed into NAAD by NAMN trans ferase (NMNAT). Three forms of this enzyme (NMNAT1, -2, and -3) have distinct subcellular localizations. The process is completed by the transformation of NAAD into NAD + by NAD + synthase (NADS). Second, the *de novo* synthesis pathway of NAD from tryptophan occurs through the kinurenine pathway (5). The first step in this pathway is the rate-limiting conversion of tryptophan to N-formylkinurenine (N-formylkin) by either IDO or TDO. Formylkinurenine is transformed into L-kinurenine (L-kin), 3-hydroxykinurenine, and 3-hydroxyanthranilic acid (3-HAA) and finally to ACMS. This compound can spontaneously condense and rearrange into quinolinic acid, which is transformed into NAMN, at which point it converges with the Preiss-Handler pathway. ACMS can also be decarboxylated into AMS by ACMS decarboxylase (ACMSD), leading to its oxidation into acetyl-CoA via the TCA cycle. Third, the NAD + salvage pathway recycles the nicotinamide generated as a by-product of the enzymatic activities of NAD + -consuming enzymes: sirtuins, PARPs, and the cADPR synthases (CD38 and CD157). Initially, NAMPT recycles nicotinamide into NMN, which is then converted into NAD + via the different NMNATs. Notes: Verdin, E., 2015. NAD⁺ in aging, metabolism, and neurodegeneration. Science. 350: 1,208–1,213. Reprinted with permission from references. Copyright 2021, AAAS.

NMN is a metabolic intermediate of the coenzyme NAD+ in the body. NAD+, as an essential coenzyme in the human body, participates in thousands of reactions within cells. However, with age and other factors, the level of NAD+ in the human body will gradually decrease, which may lead to a series of health problems. The human body mainly supplements 85% of NAD + through the Salvage synthesis pathway, and the Denovo and Preiss Handler pathways are also *in vivo* synthesis pathways for NMN (Chini et al., 2017; Li et al., 2022). In the Preiss Handler pathway, niacin (NA) is catalytically converted to nicotinic acid mononucleotide (NAMN). In the Denovo pathway, tryptophan (Trp) is converted to quinoline acid (QA), followed by the generation of NAMN. These two paths intersect at the nicotinic acid mononucleotide (NAMN) point, ultimately transforming into nicotinamide adenine dinucleotide (NAAD) and NAD+. In this way, organisms ensure a stable supply of NAD + to support their various important physiological functions through different synthetic pathways. The generation and metabolic pathways of NMN and NAD+ in the human body are shown in Figure 2.

## 4 Pharmacological effects of NMN

### 4.1 Anti-aging action

**FIGURE 3:**
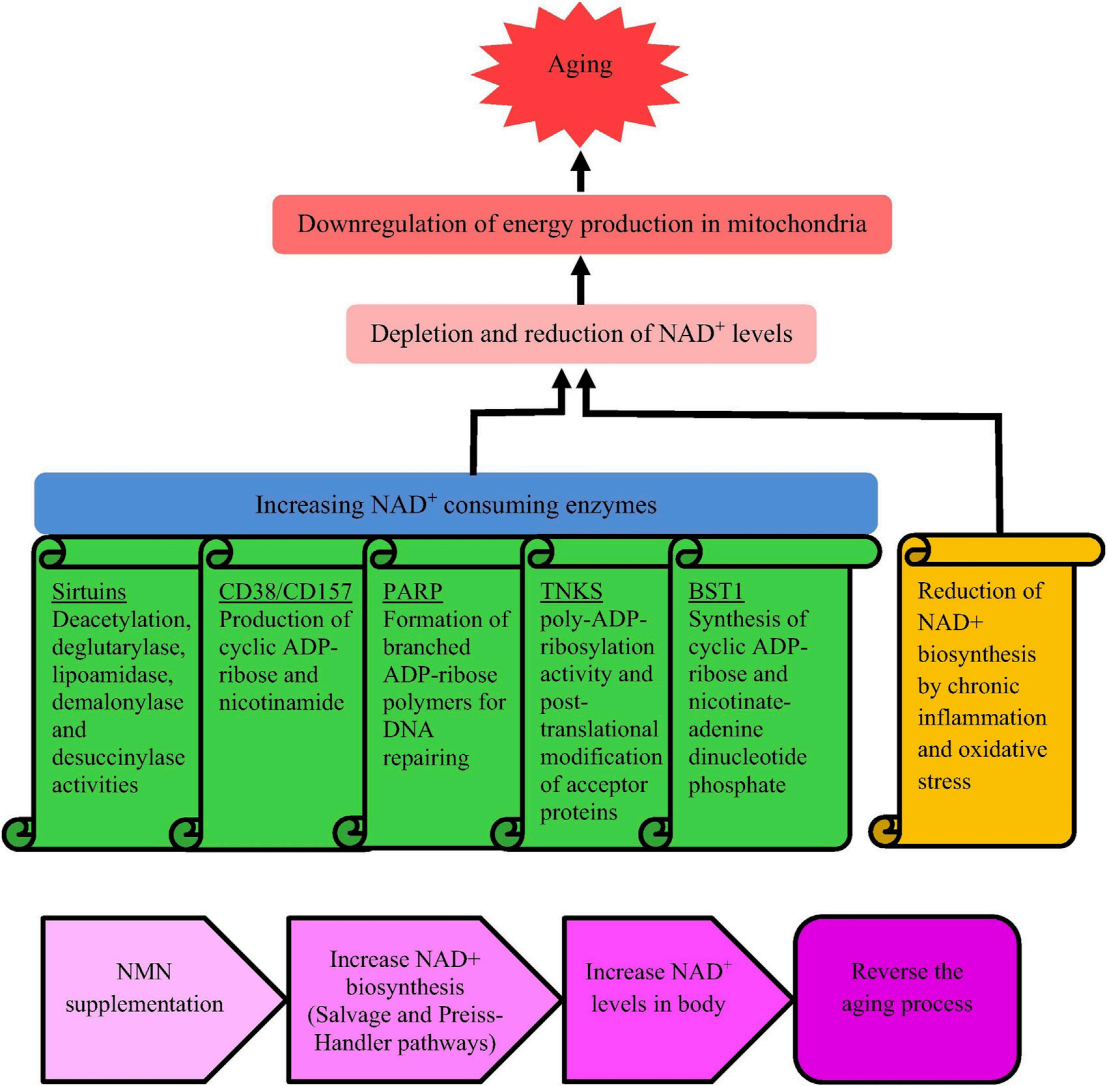
Causes for reducing NAD + levels when aging and mechanism underlying anti-aging activity of NMN. Notes: Nadeeshani, H, Li, J, Ying, T., Zhang, B, and Lu, J, 2021. Nicotinamide mononucleotide (NMN) as an anti-aging health product - promises and safety concerns. J Adv Res. 37: 267–278. Reprinted with permission from references. Copyright 2021, ELSEVIER.

Aging is a natural process, and with the consumption of NAD+, the energy of organ mitochondria decreases (Nadeeshani et al., 2021). NAD+ is crucial for cellular energy metabolism and repair, and its reduction can impair mitochondrial function and accelerate aging. Aging is characterized by decreased mitochondrial function, DNA damage, cognitive decline, osteoporosis, immune deficiency, etc. Supplementing with NAD + can alleviate these symptoms (Wang and Guo, 2015). Research has shown that NAD + precursors (NMN and NR) can maintain the number of melanocyte stem cells, enhance function, prolong mouse lifespan, and open up new directions for anti-aging science (Zhang et al., 2016). NAD+ and its precursors show potential in extending lifespan. The reasons for the decrease in NAD + levels during the aging process and the anti-aging mechanism of NMN are shown in Figure 3.

However, there are also some potential limitations and conflicting results in the anti-aging research of NMN. Firstly, NMN products produced by different brands and research institutions may have differences in purity and content, leading to inconsistencies in research results. Secondly, there is no unified standard for the dosage and usage of NMN, and there may be differences in dosage and usage in different studies, which can affect the research results. Besides, the long-term safety and efficacy of NMN still needs further research and validation, especially regarding potential adverse reactions and drug interactions that may occur when used at high doses. More importantly, aging is a complex process that involves changes in multiple physiological systems, and a single substance (such as NMN) can not comprehensively solve all problems. Therefore, combining other methods, such as a healthy diet, moderate exercise, and good lifestyle habits, is necessary to enhance the body’s anti-aging ability.

### 4.2 Improving glucose and lipid metabolism

Currently, type 2 diabetes has generally become a metabolic health problem around the world. One of its hallmark features is insulin resistance caused by increased oxidative stress, inflammation, and lipid metabolism disorders. When pancreatic β-cell dysfunction or inability to secrete sufficient insulin, it will cause insulin deficiency and eventually lead to type II diabetes (Brown et al., 2021). Pancreatic β cells are susceptible to the decrease of NAD + concentration. Once the level of NAD + decreases, it may interfere with the metabolic activity of these cells, resulting in a reduction of insulin production, which in turn increases the possibility of diabetes (Oleson et al., 2019). Studies have shown that NMN supplementation can promote insulin release from pancreatic β cells and increase the responsiveness of peripheral tissues to insulin, effectively improve diabetes caused by age and poor eating habits, and glucose tolerance and insulin sensitivity observed in elderly mice, opening up a new method for the treatment and management of diabetes (Mills et al., 2016; Sayers et al., 2020). At the same time, NMN can also regulate the metabolism of adipose tissue, promote lipolysis and its oxidation process, reduce body fat accumulation, and alleviate fat antagonism to insulin (Nahle et al., 2021). Additionally, long-term intake of NMN can increase the concentration of NAD+ in the body, which helps to improve the energy metabolism efficiency of mitochondria, thereby enhancing the body’s energy consumption ability, reducing fat accumulation, and improving the overall metabolic rate, ultimately helping to maintain a healthy weight (Stromsdorfer et al., 2016; Turner et al., 2021).

### 4.3 Improving neurological diseases

Amyloid β protein (Aβ) plays a central role in the pathogenesis of Alzheimer’s Disease (AD) and is widely considered to be the main neurotoxic substance leading to AD (Scheltens et al., 2021). The abnormal accumulation of these oligomers in the brain can lead to neuronal dysfunction and cell death, which can lead to the typical symptoms of AD, such as memory loss, cognitive impairment and so on. Some scholars have found that NMN can restore the cognition of AD model rats and is produced by increasing neuronal survival, improving energy metabolism and reducing ROS accumulation, thereby improving the cognition and memory function of AD rats caused by Aβ1-42 oligomers and restoring NAD+ and ATP levels (Wang et al., 2016). Besides, some researchers have found that NMN can effectively inhibit the production of Aβ in AD mice, reduce a load of amyloid plaques in the nervous system, synaptic damage and inflammatory response, and then improve the behavioural and cognitive impairment of AD mice (Yao et al., 2017). The above studies also show that NMN has great potential in treating AD.

Parkinson’s disease (PD) is a chronic neurological disease characterized by the degeneration and death of dopaminergic neurons in the substantia nigra, leading to motor dysfunction (Morris et al., 2024). The production of dopamine within the substantia nigra necessitates the involvement of NAD+. Consequently, any decrease in the level of NAD + will inevitably impact its synthesis process. However, studies have found that the expression of nicotinamide nucleotide adenylyltransferase (NMNAT) is decreased in the brain tissue of PD patients, and NMNAT is a key enzyme involved in NAD + biosynthesis (Challa et al., 2021; Schöndorf et al., 2018). Therefore, how to promote the synthesis of dopamine in the substantia nigra of PD requires more support from NAD+ and NMNAT to help alleviate PD. Some scholars have found that NMN can reduce apoptosis and improve energy metabolism in PD cell models, thereby restoring the intracellular levels of NAD+ and ATP in cells (Lu et al., 2014). In addition, some researchers also found that NMNAT and NAD+ were significantly weakened in PD cells. However, after NMN supplementation, not only did malondialdehyde and lactate dehydrogenase decrease in PD cells but NMNAT and NAD + also increased considerably. Furthermore, the activity of silent information regulator 1 (SIRT1) was increased, indicating that NMN treatment-activated SIRT1 may represent a new treatment strategy for PD (Zou et al., 2016).

Although NMN is believed to improve neurological function by increasing NAD + levels, its specific mechanism of action is not yet fully understood. This limits the in-depth understanding and optimization of its application in neurological diseases. Meanwhile, there are differences in the reported effectiveness of NMN in neurological diseases among different studies. Some studies suggest that NMN can significantly improve neurological function, while others may draw opposite or neutral conclusions. We believe this difference may be related to factors such as study design, sample size, and disease types. The scope of indications for NMN in neurological diseases is currently unclear. Different studies may focus on various neurological disorders, making comparing and integrating the results difficult.

### 4.4 The role of anti-vascular disorders

Vascular dysfunction refers to the functional lesions of blood vessels and nerves caused by various reasons in addition to organ damage. Blood mainly includes cardiovascular disease, cerebrovascular disease and peripheral vascular disease. Studies have shown that vascular disorders are primarily due to the imbalance between the oxidation and antioxidant systems after aging, and the accumulation of superoxide in blood vessels causes oxidative damage to the body (Conran et al., 2018; Picciotto et al., 2016. found that NMN supplementation can alleviate vascular oxidative stress and improve arteriosclerosis and functional impairment; moreover, NMN reduces vascular collagen, increases arterial elastin, reduces arteriosclerosis, and delays arterial aging; this demonstrates the potential role of NMN in protecting vascular health (Picciotto et al., 2016; Tarantini et al., 2019). Besides, NAD + plays an important role in many aspects of cardiac energy metabolism, redox reaction and cell signal transduction. When the level of NAD + decreases, the energy metabolism in cardiac cells may be affected, resulting in insufficient energy supply to cardiomyocytes (Patgiri et al., 2020) and in a mouse model of cardiomyopathy, intraperitoneal injection of NMN significantly increased NAD + levels in cardiac mitochondria and activated NAD + -dependent enzyme SIRT3, thereby improving heart and energy metabolism and promoting cardiac function recovery (Martin et al., 2017). Furthermore, NMN also has an improved effect on cerebral hemorrhage (Keep et al., 2012). Cerebral hemorrhage is another neurological disease, of which about 10%–15% of patients will further develop into a stroked state due to cerebral hemorrhage. Some scholars have found that NMN treatment significantly reduced brain edema, brain cell death, oxidative stress, neuroinflammation, microglia activation and neutrophil infiltration in the intracerebral hemorrhage area after NMN treatment in intracerebral hemorrhage model mice and successfully improved the symptoms of intracerebral hemorrhage (Wei et al., 2017).

### 4.5 Therapeutic effect of visual degenerative diseases

The causes of visual impairment are numerous and complex; however, the cell death of photoreceptors is a common fate that leads to various visual loss and even blindness diseases. Lin et al. found that the mouse model of retinal dysfunction showed early NAD + deficiency, which affected the tricarboxylic acid cycle, glycolysis and SIRT3 activity; the deficiency of NAD + leads to metabolic dysfunction and inability to cope with metabolic stress, leading to photoreceptor death and retinal degeneration (Lin et al., 2016). It can restore mice’s glycolysis, mitochondrial function and metabolic stress adaptability by supplementing NMN, thereby reducing photoreceptor cell death and significantly improving vision. These findings support NMN as a potential means of treating retinal degenerative diseases and provide a unified therapeutic target and effective therapeutic approach for ophthalmic diseases.

### 4.6 Acute kidney injury and acute lung injury

Acute kidney injury and acute lung injury are frequently encountered serious medical conditions. The incidence of acute kidney injury and acute lung injury is related to many factors, but the mortality of both is high. With the increase of age, the levels of SIRT1 and NAD+ in organisms will gradually decrease; significantly, the decrease of SIRT1 and NAD+ in the kidneys of elderly organisms will lead to an increase in the incidence of acute kidney injury. In a model of cisplatin-induced acute kidney injury in mice, it was found that the protective group of mice could resist cisplatin-induced acute kidney injury by supplementing NMN in advance. Endogenous NAD+ was considered a potential therapeutic target for acute kidney injury in older patients, and NMN supplementation was an excellent therapeutic strategy (Guan et al., 2017). In addition, studies have found that NMN has a protective effect on sepsis-induced lung injury in mice. In the lung tissue of mice with lung injury induced by early use of NMN, the myeloperoxidase (MPO) was significantly reduced. The levels of NAD+ and ATP were significantly increased, and the expression of SIRT1 was upregulated. The acetylation and phosphorylation of NF-κB-p65 were inhibited, thereby protecting the NMN group mice from sepsis-induced lung injury (He et al., 2024). The team believes that NMN promotes macrophage polarization through the SIRT1/NF-κB pathway to prevent sepsis-induced acute lung injury, which may be an effective strategy for preventing or treating sepsis-induced acute lung injury.

### 4.7 Protection against blood failure

Hematopoietic stem cell (HSC) is a special cell existing in bone marrow, which has the ability to self-renew and differentiate into various types of blood cells, such as red blood cells, white blood cells and platelets. In recent years, studies have shown that increased oxidative phosphorylation reflected by increased mitochondrial activity, coupled with impaired mitochondrial stress response, can seriously damage HSC regeneration (Hu et al., 2023). In an animal experiment to explore NAD + -stimulated hematopoiesis, it was found that supplementation of NAD + enhancer (NMN) could increase mitochondrial clearance in hematopoietic-impaired mice, thereby reducing mitochondrial activity in HSC, leading to increased asymmetric division of HSC and promoting the recovery of hematopoietic function (Vannini et al., 2018). In addition, some scholars have found that the number of white blood cells and bone marrow nucleated cells in peripheral blood of mice increased significantly after NAD+ was given to mice after radiation injury, and the apoptosis of bone marrow cells decreased, and the immunity of mice after radiation was improved (Tan et al., 2010). Therefore, supplementing with NMN to promote the recovery of hematopoietic function and resist various causes of blood failure may be a new treatment strategy.

## 5 Human clinical trials of NMN

Currently, several human clinical trials on NMN have been carried out at home and abroad. The supplemental dose of NMN is 100–1,250 mg/d, and there are no adverse reactions, indicating that it has good human tolerance and high safety. A majority of human clinical trials involving NMN have been conducted between 2020 and 2024, encompassing treatment durations ranging from a minimum of 3 weeks to a maximum of 12 weeks. These trials span numerous countries, including the United States, Japan, China, and India (Fukamizu et al., 2022; Gao et al., 2023; Igarashi et al., 2022; Irie et al., 2020; Kuerec et al., 2024; Liao et al., 2021; Yamaguchi et al., 2024; Yi et al., 2023; Yoshino et al., 2021). In addition, the subjects were mainly middle-aged healthy men and women, but there were also some other subjects and patients, such as athletes, overweight or obese postmenopausal pre-diabetic women and insomnia patients. In the following clinical studies, the levels of NAD+ in the blood of subjects and patients significantly increased after oral administration of NMN, and NMN dose dependence was shown. Moreover, according to the different contents of each NMN clinical trial project, other data also changed significantly, including a significant increase in skeletal muscle insulin signal (Akt and mTOR phosphorylation) in overweight or obese postmenopausal women with prediabetes, and lactate dehydrogenase, dimethylpyridine and tetramethylpyridine, 6-min walking test distance, and SF-36 scores in adult healthy men and women. Significantly improved, but their albumin-to-globulin ratio and fasting blood glucose were significantly reduced. For elderly healthy men, there is also a significant improvement in their left-hand grip strength and gait speed; the first and second anaerobic thresholds, maximum oxygen uptake, oxygen pulse, and running economy of athletes have significantly increased; however, a multicenter controlled clinical trial on the treatment of chronic insomnia after oral NMN has not yielded detailed research results, which require further observation (trial registration: China Clinical Trial Registration Center (chictr. org. cn) ChiCTR220058001) (Gao et al., 2023).

However, in human trials, the effect of NMN is not as significant and effective as in animal experiments. For instance, in animal experiments, especially studies on aging or obese mice, NMN is proven to significantly increase the level of NAD+ in skeletal muscle, thus improving mice’s body weight and glucose and lipid metabolism. When this intervention was transferred to human trials, similar significant effects did not occur. This difference suggests that the human body may have a more powerful NAD + regulatory mechanism to maintain its homeostasis; this mechanism enables the NAD + levels in many tissues and organs to remain within a relatively constant range; therefore, even with exogenous supplementation of NAD + precursors such as NMN, the NAD + homeostasis in the human body will not be easily altered (Chen et al., 2023; Khaidizar et al., 2021). In addition, the dosage of NMN in clinical trials needs to be considered. For example, the dosages (including 100 mg/d, 250 mg/d, or 300 mg/d) are likely too low for the human body to produce the significant effects observed in animal experiments. Furthermore, combined with the individual and physiological differences between human and animals, this further explains why the effect of NMN in human experiments is not as significant as in animal experiments. Therefore, in future human studies, it may be necessary to consider using higher doses to explore the impact of NMN on human NAD + levels and related metabolic indicators. In recent years, the main results of human clinical trials on NMN are summarized in Table 1.

**TABLE 1:** Summary of human clinical trials of NMN.

Authors	Subjects	Number of participants	NMN and control group	Treatment cycle	Major results
Yoshino	Overweight or obese postmenopausal women with prediabetes	13	250 mg/d	10 weeks	Skeletal muscle insulin signaling (phosphorylation of Akt and mTOR)↑, Blood NAD+ ↑, There were no significant changes in body weight, body fat percentage, blood lipid and blood glucose in the participants
		12	Placebo		
Fukamizu	Healthy adult men and women aged 20–65 years	16	1,250 mg/d	4 weeks	Lactate dehydrogenase ↑, albumin to globulin ratio ↓, there was no significant difference in hematology, clinical biochemistry and urine test
		15	Placebo		
Irie	Healthy men aged 40–60 years	10	100 mg/d/w	3 weeks	Blood dimethylpyridine and tetramethylpyridine ↑, there were no significant changes in the subjects’ heart rate, blood pressure, blood oxygen saturation, body temperature, and other symptoms
		10	250 mg/d/w		
		10	500 mg/d/w		
Yamaguchi	Healthy middle-aged men	12	125 mg/d	8 weeks	NAD+ in peripheral blood mononuclear cells ↑, fasting insulin and fasting blood glucose ↓, there was no significant change and improvement in the sleep quality of the participants
		13	Placebo		
Yi	Healthy adult middle-aged men and women	20	300 mg/d	60 days	Plasma NAD+ ↑, 6-min walk test distance ↑, SF-36 score ↑, HOMA-IR had no significant change
		20	600 mg/d		
		20	900 mg/d		
		20	Placebo		
Gao	Patients with chronic insomnia	200	300 mg/d	60 days	Unknown
		200	Placebo		
Liao	Young and middle-aged leisure training athletes	12	300 mg/d	6 weeks	The first anaerobic threshold and the second anaerobic threshold ↑, maximum oxygen uptake, oxygen pulse, running economy ↑
		12	600 mg/d		
		12	1,200 mg/d		
		12	Placebo		
Igarashi	Healthy elderly men	21	250 mg/d	12 weeks	Left hand grip strength ↑, gait speed ↑, right hand grip strength, standing test, skeletal muscle mass, insulin sensitivity and cognitive function did not improve
		21	Placebo		
Kumbhar	Healthy male adults	20	300 mg/d	60 days	Plasma NAD+ ↑, there was no significant change in 6-min walk test distance, SF-36 score and HOMA-IR.
		20	600 mg/d		
		20	900 mg/d		
		20	Placebo		

At present, research on NMN mainly focuses on animal models and *in vitro* experiments, which provide us with a preliminary understanding of the possible biological effects and mechanisms of action of NMN. However, generalizing these findings to humans still faces challenges as human physiological and pathological processes differ from those in experimental animals, affecting the clinical application of NMN. Therefore, we need more high-quality clinical studies to validate the efficacy and safety of NMN in humans. In addition, we have found that many human clinical trials related to NMN currently encounter problems such as small sample sizes, subject selection bias, short follow-up periods, and inconsistent research results. Therefore, we believe that researchers can seek possible solutions in the future through the following points: First, strengthening cooperation, establishing multi-center, cross-border cooperation networks, uniting medical and research institutions, and expanding recruitment scope to increase sample size. This helps to improve sample diversity and research efficiency. Secondly, data sharing: promoting data sharing among research institutions, utilizing large databases to validate NMN research results, and enhancing statistical capabilities and generalizability of conclusions. Subsequently, innovative recruitment and incentive measures were implemented: raising public awareness through social media, patient organizations, etc., designing economic compensation and other incentive measures to increase participation willingness. Afterwards, standardized research methods will be established: adopt internationally recognized research methods and standards to improve the reproducibility and comparability of experiments. Again, extending follow-up time: When designing the study, consider long-term observation, optimize follow-up strategies, and ensure complete and accurate data. Finally, interdisciplinary collaboration: promoting collaboration among multiple disciplines such as biology, medicine, and pharmacy to jointly advance clinical research on NMN and accelerate its clinical application process.

## 6 Applications of NMN

### 6.1 Application of NMN in medicine

Given the various potential biological activities of NMN, the current medical field has shown great interest in developing drugs with NMN as the key active ingredient, which has become an important research direction and hotspot. Various compositions have emerged in the market, including NAD+, NMN, NR, and other components with significant biological activity. These compositions are designed for anti-aging and antioxidant therapy, aiming to help people delay aging and protect cells from oxidative stress damage. In addition, drugs or health products with NMN as the main activity are mainly sold in the United States, Japan, Australia and other countries. According to the research and statistics of the QYResearch team, the global NMN market sales reached two billion yuan in 2023 and is expected to reach 4.8 billion yuan by 2030, with a compound annual growth rate of 12.6% (2024–2030). Meanwhile, the market size of NMN healthcare products in China will climb to 27.013 billion yuan in 2023. Currently, brands such as McKinley in the United States, RevitaLife in the United States, High Grade Labo in Japan, Swisse in Australia, Emerging in Japan, ASHOKO in Japan, Hermetin in Germany, GeneHarbor in Chinese Hong Kong, Wright Life in Chinses Hong Kong have all launched NMN-containing healthcare products, all focusing on anti-aging and life extension effects.

In recent years, American scientist Huizenga has successfully developed a composition integrating key active ingredients such as NAD+, NMN, and NR. The design of this composition aims at anti-aging and antioxidant treatment, and through its unique combination of ingredients, it aims to help the human body resist the aging process and effectively protect cells from damage caused by oxidative stress. Professor Imai from Washington University has developed a new type of treatment method specifically targeting age-related obesity, hyperlipidemia, and type 2 diabetes. The core of this treatment lies in using NMN as an active ingredient, aiming to improve these age-related health issues through its unique biological activity. In China, the famous scientist Rongzhao Fu and his team have carried out in-depth exploration in many fields. They have successfully developed a drug with NMN as a key active ingredient, which is specifically used to treat arteriosclerosis and cardiovascular diseases, aiming to improve the symptoms and processes of these diseases through the biological effects of NMN. They also invented a drug that simultaneously added Nicotinamide Adenine Dinucleotide Hydrate (NADH) and NMN to treat PD, aiming to bring new therapeutic hope to patients with PD through the synergistic effect of these two active ingredients. At the same time, they have also developed an anti-aging beauty skin care composition containing NMN, which helps the skin to stay young and reduces the formation of wrinkles and fine lines through the anti-aging properties of NMN. Professor Miao chaoyu from the Naval Medical University of Chinese PLA utilizes NMN to prepare drug compositions to promote nerve regeneration after cerebral ischemia and provide new therapies for brain health. Therefore, the application of NMN in the field of medical and healthcare at home and abroad is becoming increasingly widespread. It not only shows potential in diseases such as atherosclerosis, obesity, diabetes, and PD, but also achieves remarkable results in anti-aging, beauty, and skin care, providing more possibilities for people’s health and quality of life.

### 6.2 Application of NMN in Traditional Chinese medicine

Traditional Chinese medicine has been a traditional medicine in China for many years, and its unique compatibility and treatment methods are of great significance for maintaining human health and treating diseases. Some scholars have recently found that some traditional Chinese medicines are also rich in NMN and NAD+. Modern research has found that Dendrobium officinale has anti-aging, immune enhancement, and hypoglycemic effects, which are very similar to the functions of NMN and NAD+. Therefore, Liu et al. used UPLC-MS/MS detection technology to detect NMN and NAD+ in Dendrobium officinale and found that Dendrobium officinale not only contains NMN and NR but also a large amount of NAD+, which is three times the content of NMN (Liu et al., 2021). This study is also the first to establish a simultaneous determination method for NMN and NAD + in Dendrobium officinale by liquid chromatography-mass spectrometry. This method has simple sample pretreatment, fast detection speed, high accuracy, and wide applicability. In addition, another type of traditional Chinese medicine (Tougu Cao) contains multiple polysaccharides and active ingredients, which relax tendons, promote blood circulation, reduce swelling and pain, and dispel wind and dampness (Li et al., 2021). Although Tougucao itself does not directly contain NMN, some components in its extract may help to promote the synthesis of NMN or increase the activity of NAD+. Similar traditional Chinese medicines that can promote the synthesis of NMN or increase the activity of NAD + contain curcumin, mangosteen peel, notoginseng stems and leaves, etc., and traditional Chinese medicine monomers also include ginsenosides, quercetin, astragaloside and Lycium barbarum polysaccharides (Guo et al., 2023; Liu et al., 2017; Tang et al., 2022; Xie et al., 2023; Yang et al., 2019; Zhou et al., 2023).

### 6.3 Application of NMN in food

As a natural compound, NMN is widely distributed in various foods, including vegetables, certain fungi, meat products, and seafood such as shrimps. The presence of NMN can be detected, as shown in Table 2 (Mills et al., 2016). Currently, some scholars have quantified NR, NMN and NAD+ in milk by fluorescence enzyme-linked method and found that these three substances exist in human and donkey milk and are selectively distributed in other milk, among which human milk is the most abundant source of NMN (Ummarino et al., 2017). Other researchers have detected NMN from pig placenta extract and found that pig placenta can be applied to the reconstruction of human epidermal stratum corneum and can significantly improve the induced NAD + consumption, thereby increasing skin NAD + levels (Katayoshi et al., 2022). Megumi has successfully developed a food composition containing NMN and resveratrol in Japan; resveratrol is a natural polyphenolic compound with estrogenic effects, exhibiting strong antioxidant and antibacterial properties; it can also inhibit tyrosinase activity, improve metabolic syndrome, and prolong lifespan (Zhao et al., 2018). The experimental results show that this combination can effectively reduce the blood’s total cholesterol and low-density lipoprotein content, reduce the risk of myocardial infarction, and reduce the content of neutral fat such as uric acid and triglyceride. Besides, according to the “Announcement on the Acceptance of New Food Additives on 28 January 2023”released by the Government Service Platform of the National Health Commission of China, NMN has been officially accepted as a new variety of food additives. This measure means that NMN’s status as a food additive has been preliminarily recognized by the government, opening the door for further research and application in the food industry. Although the application of NMN in China is mainly concentrated in the medical field and the application in the food field is still blank, with the deepening understanding of NMN safety and the support of national policies, there will be more food or health products containing NMN in the future, which will bring more benefits to people’s healthy life.

**TABLE 2:** Content of NMN in various natural foods.

Food types	Food names	NMN content (mg/100 g)
Vegetables	Broccoli	0.25–1.12
	Cucumber seeds	0.56
	Edamame	0.47–1.88
	Yellow-flowered peel	0.65
	Cabbage	0.0–0.9
Fruits	Avocado	0.36–1.6
	Tomato	0.26–0.30
Meat	Raw beef	0.06–0.42
Seafood	Shrimp	0.22
Other	Mushrooms	0.0–1.01

## 7 Prospects

NMN is a substance that has received much attention in the field of anti-aging in recent years. It is also an important precursor of coenzyme NAD+ in the human body, and NAD + plays a key role in maintaining cellular energy metabolism, DNA repair, and regulating aging-related gene expression. First, NMN has enormous potential in anti-aging, as it can effectively enhance NAD + levels in the body, thereby improving various age-related physiological decline, such as cardiovascular, neurological, and metabolic functions. Another advantage of NMN is its safety, as it has good safety and tolerability in animal models and high safety in the human body without significant side effects. Finally, NMN is also easily absorbed, and compared to other NAD + precursors, NMN can directly enter cells and convert to NAD + without going through complex metabolic pathways.

However, NMN also has some limitations. First of all, the effectiveness of NMN varies significantly among individuals: there are significant differences in the response of different populations to NMN, which may be related to factors such as genetic background, age, and lifestyle. Secondly, there is a lack of high-quality clinical evidence: Currently, most research on NMN focuses on animal models, with relatively few human clinical trials and limited scale, making it difficult to comprehensively evaluate its actual benefits and long-term safety for human health. Subsequently, the cost was high: currently, the price of NMN supplements is relatively high, which limits their popularity among ordinary consumers. Lastly, regulatory limitations: In some countries and regions, there is still legal uncertainty regarding the sale of NMN as a food or health product.

Based on the above issues, our team unanimously believes that NMN must be further enhanced in the following aspects. Firstly, strengthen the basic research of NMN; although we have a particular understanding of some biological activities of NMN, its specific mechanism of action in cells and its interaction with other molecules still need to be further studied. This will help us to understand the mechanism of NMN more comprehensively and provide more powerful support for its application in anti-aging, diabetes, AD, PD and other fields. Secondly, reduce the production cost of NMN. The production cost of NMN is relatively high, which limits its popularity in the market at present. Therefore, we can reduce the production cost of NMN by improving production processes, increasing production efficiency, and other methods, thereby making it more cost-effective. Thirdly, high-quality clinical human trials across multiple centers should be conducted. The current clinical trials have low quality due to various factors, such as short follow-up periods, small sample sizes, differences in sample selection, and inconsistent research results. Furthermore, strengthening the supervision and verification of NMN products is also essential. Due to the lack of strict supervision and verification of NMN products in the current market, there are certain risks to their safety and effectiveness. Hence, we need to strengthen the supervision and verification of NMN products to ensure their quality and safety. This will help protect consumer rights and promote the healthy development of the NMN market. With the continuous development of technology, we believe that more research will reveal the potential of NMN in treating or protecting various diseases. In addition to being mainly used as health products and food, NMN can also be combined with drug development to develop more treatment methods for age-related diseases.

## 8 Conclusion

To summarize, the potential and value of NMN are enormous, transcending current limitations and challenges. Its unique properties suggest profound implications for the pharmaceutical industry. With ongoing research refining our understanding of its mechanisms and applications, NMN is poised to become a key player in drug development and health management. Its versatility in treating various conditions and enhancing overall wellness promises a brighter future for both scientific research and human health.

## References

[B1] Akbari S. Kariznavi E. Jannati M. Elyasi S. (2020). Curcumin as a preventive or therapeutic measure for chemotherapy and radiotherapy induced adverse reaction: a comprehensive review. Food Chem. Toxicol. 145, 111699. 10.1016/j.fct.2020.111699 32858134

[B2] Alegre G. F. S. Pastore G. M. (2023). NAD+ precursors nicotinamide mononucleotide (NMN) and nicotinamide riboside (NR): potential dietary contribution to health. Curr. Nutr. Rep. 12, 445–464. 10.1007/s13668-023-00475-y 37273100 PMC10240123

[B3] Brown M. R. Holmes H. Rakshit K. Javeed N. Her T. K. Stiller A. A. (2021). Electrogenic sodium bicarbonate cotransporter NBCe1 regulates pancreatic β cell function in type 2 diabetes. J. Clin. Invest 131, e142365. 10.1172/JCI142365 34623331 PMC8409580

[B4] Challa S. Khulpateea B. R. Nandu T. Camacho C. V. Ryu K. W. Chen H. (2021). Ribosome ADP-ribosylation inhibits translation and maintains proteostasis in cancers. Cell 184, 4531–4546.e26. 10.1016/j.cell.2021.07.005 34314702 PMC8380725

[B5] Chen T. Cao H. Dong L. Ji Z. Cao J. (2023). Research progress on the effects of β-nicotinamide mononucleotides on physiological functions. Food Sci. 44, 382–391. 10.7506./.spkx1002-6630-20220713-139

[B6] Chini C. C. S. Tarragó M. G. Chini E. N. (2017). NAD and the aging process: role in life, death and everything in between. Mol. Cell Endocrinol. 455, 62–74. 10.1016/j.mce.2016.11.003 27825999 PMC5419884

[B7] Conran N. Silva J. A. F. Gotardo E. M. F. Chweih H. Costa F. F. Leonardo F. C. (2018). The ribonucleotide reductase inhibitor, didox, reduces the *in vivo* vascular inflammation and oxidative stress induced by acute hemolysis. Blood 132, 1034. 10.1182/blood-2018-99-119224

[B8] Duan R. Li Y. Zhang R. Hu X. Wang Y. Zeng J. (2023). Reversing acute kidney injury through coordinated interplay of anti-inflammation and iron supplementation. Adv. Mater 35, e2301283. 10.1002/adma.202301283 37029662

[B9] Fukamizu Y. Uchida Y. Shigekawa A. Sato T. Kosaka H. Sakurai T. (2022). Safety evaluation of β-nicotinamide mononucleotide oral administration in healthy adult men and women. Sci. Rep. 12, 14442. 10.1038/s41598-022-18272-y 36002548 PMC9400576

[B10] Gao X. Li J. Xu S. Li X. Wang X. Li Y. (2023). Oral nicotinamide mononucleotide (NMN) to treat chronic insomnia: protocol for the multicenter, randomized, double-blinded, placebo-controlled trial. Trials 24, 340. 10.1186/s13063-023-07351-8 37202819 PMC10193321

[B11] Guan Y. Wang S. R. Huang X. Z. Xie Q. H. Xu Y. Y. Shang D. (2017). Nicotinamide mononucleotide, an NAD+ precursor, rescues age-associated susceptibility to AKI in a sirtuin 1-dependent manner. J. Am. Soc. Nephrol. 28, 2337–2352. 10.1681/ASN.20160.40385 28246130 PMC5533221

[B12] Guo C. Huang Q. Wang Y. Yao Y. Li J. Chen J. (2023). Therapeutic application of natural products: NAD+ metabolism as potential target. Phytomedicine 114, 154768. 10.10.16/j.phymed.2023.154768 36948143

[B13] He S. Jiang X. Yang J. Wu Y. Shi J. Wu X. (2024). Nicotinamide mononucleotide alleviates endotoxin-induced acute lung injury by modulating macrophage polarization via the SIRT1/NF-κB pathway. Pharm. Biol. 62, 22–32. 10.1080/13880209.20.23.2292256 38100537 PMC10732210

[B14] Hu M. Chen N. Chen M. Chen F. Lu Y. Xu Y. (2023). Transcription factor Nkx2-3 maintains the self-renewal of hematopoietic stem cells by regulating mitophagy. Leukemia 37, 1361–1374. 10.1038/s41375-023-01907-y 37095209

[B15] Igarashi M. Nakagawa-Nagahama Y. Miura M. Kashiwabara K. Yaku K. Sawada M. (2022). Chronic nicotinamide mononucleotide supplementation elevates blood nicotinamide adenine dinucleotide levels and alters muscle function in healthy older men. NPJ Aging 8, 5. 10.1038/s41514-022-00084-z 35927255 PMC9158788

[B16] Irie J. Inagaki E. Fujita M. Nakaya H. Mitsuishi M. Yamaguchi S. (2020). Effect of oral administration of nicotinamide mononucleotide on clinical parameters and nicotinamide metabolite levels in healthy Japanese men. Endocr. J. 67, 153–160. 10.1507/endocrj.EJ1.9-0313 31685720

[B17] Jiang Y. Deng Y. Pang H. Ma T. Ye Q. Chen Q. (2022). Treatment of SARS-CoV-2-induced pneumonia with NAD+ and NMN in two mouse models. Cell Discov. 8, 38. 10.1038/s41421-022-00409-y 35487885 PMC9053567

[B18] Katayoshi T. Yamaura N. Nakajo T. Kitajima N. Tsuji-Naito K. (2022). Porcine placental extract increase the cellular NAD levels in human epidermal keratinocytes. Sci. Rep. 12, 19040. 10.1038/s41598-022-23446-9 36352014 PMC9646745

[B19] Keep R. F. Hua Y. Xi G. (2012). Intracerebral haemorrhage: mechanisms of injury and therapeutic targets. Lancet Neurol. 11, 720–731. 10.1016/S1474-4422(12)70104-7 22698888 PMC3884550

[B20] Khaidizar F. D. Bessho Y. Nakahata Y. (2021). Nicotinamide phosphoribosyltransferase as a key molecule of the aging/senescence process. Int. J. Mol. Sci. 22, 3709. 10.3390/ijms22073709 33918226 PMC8037941

[B21] Klausner G. Bensadoun R. J. Champion A. Benzaquen D. Canova C. H. Claren A. (2021). State of art of photobiomodulation in the management of radiotherapy adverse events: indications and level of evidence. Cancer Radiother. 25, 584–592. French. 10.1016/j.canrad.2021.06.025 34272181

[B22] Kuerec A. H. Wang W. Yi L. Tao R. Lin Z. Vaidya A. (2024). Towards personalized nicotinamide mononucleotide (NMN) supplementation: nicotinamide adenine dinucleotide (NAD) concentration. Mech. Ageing Dev. 218, 111917. 10.1016/j.mad.2024.111.917 38430946

[B23] Li H. Liu F. Jiang W. Wang K. Cao X. Zou J. (2022). TREM2 ameliorates lipopolysaccharide-induced oxidative stress response and neuroinflammation by promoting sirtuin3 in BV2 cells. Neurotox. Res. 40, 56–65. 10.1007/s12640-021-00459-2 35013907

[B24] Li Y. R. Xu Y. L. Du X. Y. Yang S. D. Lu L. (2021). Characterization of the complete plastid genome of Gaultheria griffithiana (Ericaceae). Mitochondrial DNA B Resour. 6, 1575–1577. 10.1080/23802359.2021.1914227 34212078 PMC8218835

[B25] Liao B. Zhao Y. Wang D. Zhang X. Hao X. Hu M. (2021). Nicotinamide mononucleotide supplementation enhances aerobic capacity in amateur runners: a randomized, double-blind study. J. Int. Soc. Sports Nutr. 18, 54. 10.1186/s12970-021-00442-4 34238308 PMC8265078

[B26] Lin J. B. Kubota S. Ban N. Yoshida M. Santeford A. Sene A. (2016). NAMPT-mediated NAD(+) biosynthesis is essential for vision in mice. Cell Rep. 17, 69–85. 10.1016/j.celrep.2016.08.073 27681422 PMC5104206

[B27] Liu B. H. Gu Y. H. Tu Y. He W. M. Wu W. Liu Y. L. (2017). Molecular regulative mechanisms of aging and interventional effects of Chinese herbal medicine. Zhongguo Zhong Yao Za Zhi 42, 3065–3071. Chinese. 10.19540/j.cnki.cjcmm.20170731.001 29171222

[B28] Liu X. Yang H. Zhao J. Meng C. Li C. Zhang D. (2021). UPLC-MS/MS was used to determine the content of nicotinamide mononucleotide and nicotinamide adenine dinucleotide in D.officinale and its closely related species. Chin. J. Traditional Chin. Med. 46, 4034–4039. 10.19540/j.cnki.cjcmm.20210507.303 34467712

[B29] Lu L. Tang L. Wei W. Hong Y. Chen H. Ying W. (2014). Nicotinamide mononucleotide improves energy activity and survival rate in an *in vitro* model of Parkinson's disease. Exp. Ther. Med. 8, 943–950. 10.3892/etm.2014.1842 25120628 PMC4113526

[B30] Martin A. S. Abraham D. M. Hershberger K. A. Bhatt D. P. Mao L. Cui H. (2017). Nicotinamide mononucleotide requires SIRT3 to improve cardiac function and bioenergetics in a friedreich's ataxia cardiomyopathy model. JCI Insight 2, e93885. 10.1172/j.ci.insight.93885 28724806 PMC5518566

[B31] Mills K. F. Yoshida S. Stein L. R. Grozio A. Kubota S. Sasaki Y. (2016). Long-term administration of nicotinamide mononucleotide mitigates age-associated physiological decline in mice. Cell Metab. 24, 795–806. 10.1016/j.cmet.2016.09.013 28068222 PMC5668137

[B32] Morris H. R. Spillantini M. G. Sue C. M. Williams-Gray C. H. (2024). The pathogenesis of Parkinson's disease. Lancet 403, 293–304. 10.1016/S0140-6736(23)01478-2 38245249

[B33] Nadeeshani H. Li J. Ying T. Zhang B. Lu J. (2021). Nicotinamide mononucleotide (NMN) as an anti-aging health product - promises and safety concerns. J. Adv. Res. 37, 267–278. 10.1016/j.jare.2021.08.003 35499054 PMC9039735

[B34] Nahle A. Joseph Y. D. Pereira S. Mori Y. Poon F. Ghadieh H. E. (2021). Nicotinamide mononucleotide prevents free fatty acid-induced reduction in glucose tolerance by decreasing insulin clearance. Int. J. Mol. Sci. 22, 13224. 10.3390/ijms222413224 34948019 PMC8709165

[B35] Oleson B. J. Broniowska K. A. Yeo C. T. Flancher M. Naatz A. Hogg N. (2019). The Role of Metabolic Flexibility in the regulation of the DNA damage response by nitric oxide. Mol. Cell Biol. 39, e00153–19. 10.1128/MCB.00153-19 31235477 PMC6712938

[B36] Ou L. Zhao X. Wu I. J. Yu Z. Xiong Z. Xia L. C. (2024). Molecular mechanism of NAD+ and NMN binding to the Nudix homology domains of DBC1. Int. J. Biol. Macromol. 262, 130131. 10.1016/j.ijbiomac.2024.130131 38354937

[B37] Patgiri A. Skinner O. S. Miyazaki Y. Schleifer G. Marutani E. Shah H. (2020). An engineered enzyme that targets circulating lactate to alleviate intracellular NADH:NAD+ imbalance. Nat. Biotechnol. 38, 309–313. 10.1038/s41587-019-0377-7 31932725 PMC7135927

[B38] Picciotto N. E. Gano L. B. Johnson L. C. Martens C. R. Sindler A. L. Mills K. F. (2016). Nicotinamide mononucleotide supplementation reverses vascular dysfunction and oxidative stress with aging in mice. Aging Cell 15, 522–530. 10.1111/acel.12461 26970090 PMC4854911

[B39] Rohrhoff N. J. McNeill D. B. Boggan J. C. (2014). An adverse reaction to a medication given to treat an adverse reaction: a teachable moment. JAMA Intern Med. 174, 1035–1036. 10.1001/jamainternmed.2014.1605 24841571

[B40] Sayers S. R. Beavil R. L. Fine N. H. F. Huang G. C. Choudhary P. Pacholarz K. J. (2020). Structure-functional changes in eNAMPT at high concentrations mediate mouse and human beta cell dysfunction in type 2 diabetes. Diabetologia 63, 313–323. 10.1007/s0012.5-019-05029-y 31732790 PMC6946736

[B41] Scheltens P. Strooper B. Kivipelto M. Holstege H. Chételat G. Teunissen C. E. (2021). Alzheimer's disease. Lancet 397 (10284), 1577–1590. 10.1016/S0140-6736(20)32.205-4 33667416 PMC8354300

[B42] Schöndorf D. C. Ivanyuk D. Baden P. Sanchez-Martinez A. Cicco S. Yu C. (2018). The NAD+ precursor nicotinamide riboside rescues mitochondrial defects and neuronal loss in iPSC and fly models of Parkinson's disease. Cell Rep. 23, 2976–2988. 10.1016/j.cel.rep.2018.05.009 29874584

[B43] Song Q. Zhou X. Xu K. Liu S. Zhu X. Yang J. (2023). The safety and anti-aging effects of nicotinamide mononucleotide in human clinical trials: an update. Adv. Nutr. 14, 1416–1435. 10.1016/j.advnut.2023.08.008 37619764 PMC10721522

[B44] Stromsdorfer K. L. Yamaguchi S. Yoon M. J. Moseley A. C. Franczyk M. P. Kelly S. C. (2016). NAMPT-mediated NAD(+) biosynthesis in adipocytes regulates adipose tissue function and multi-organ insulin sensitivity in mice. Cell Rep. 16, 1851–1860. 10.1016/j.celr.ep.2016.07.027 27498863 PMC5094180

[B45] Sun C. Liu X. Wang B. Wang Z. Liu Y. Di C. (2019). Endocytosis-mediated mitochondrial transplantation: transferring normal human astrocytic mitochondria into glioma cells rescues aerobic respiration and enhances radiosensitivity. Theranostics 9, 3595–3607. 10.7150/thno.33100 31281500 PMC6587163

[B46] Tan Y. You W. Li M. Zhang J. Zhang J. (2010). Effect of oxidized coenzyme NAD+ on hematopoietic function in mice with radiation injury. Guangdong Med. 31, 960–962. 10.13820/j.cnki.gdyx.2010.08.005

[B47] Tang K. Qin W. Wei R. Jiang Y. Fan L. Wang Z. (2022). Ginsenoside Rd ameliorates high glucose-induced retinal endothelial injury through AMPK-STRT1 interdependence. Pharmacol. Res. 179, 106123. 10.1016/j.phrs.2022.106123 35150861

[B48] Tarantini S. Valcarcel-Ares M. N. Toth P. Yabluchanskiy A. Tucsek Z. Kiss T. (2019). Nicotinamide mononucleotide (NMN) supplementation rescues cerebromicrovascular endothelial function and neurovascular coupling responses and improves cognitive function in aged mice. Redox Biol. 24, 101192. 10.1016/j.redox.2019.101192 31015147 PMC6477631

[B49] Turner J. Licollari A. Mihalcea E. Tan A. (2021). Safety evaluation for restoring® NMN, a NAD+ precursor. Front. Pharmacol. 12, 749727. 10.3389/fphar.2021.749727 34867355 PMC8632654

[B50] Ummarino S. Mozzon M. Zamporlini F. Amici A. Mazzola F. Orsomando G. (2017). Simultaneous quantitation of nicotinamide riboside, nicotinamide mononucleotide and nicotinamide adenine dinucleotide in milk by a novel enzyme-coupled assay. Food Chem. 221, 161–168. 10.1016/j.foodchem.2016.10.032 27979136

[B51] Vannini N. Campos V. Girotra M. Rojas-Sutterlin S. Naveiras O. Ragusa S. (2018). The NAD+ salvage pathway potently stimulates hematopoiesis through increased mitochondrial clearance and asymmetric division. Blood 132, 641. 10.1182/blood-2018-99-117388

[B52] Verdin E. (2015). NAD⁺ in aging, metabolism, and neurodegeneration. Science 350, 1208–1213. 10.1126/science.aac4854 26785480

[B53] Wang Q. L. Guo S. J. (2015). Sirtuins function as the modulators in aging-related diseases in common or respectively. Chin. Med. J. Engl. 128, 1671–1678. 10.4103/.0366-6999.158375 26063372 PMC4733746

[B54] Wang X. Hu X. Yang Y. Takata T. Sakurai T. (2016). Nicotinamide mononucleotide protects against β-amyloid oligomer-induced cognitive impairment and neuronal death. Brain Res. 1643, 1–9. 10.1016/j.brainres.2016.04.060 27130898

[B55] Wei C. C. Kong Y. Y. Li G. Q. Guan Y. F. Wang P. Miao C. Y. (2017). Nicotinamide mononucleotide attenuates brain injury after intracerebral hemorrhage by activating Nrf2/HO-1 signaling pathway. Sci. Rep. 7, 717. 10.1038/s41598-017-00851-z 28386082 PMC5429727

[B56] Xie W. Zhu T. Zhou P. Xu H. Meng X. Ding T. (2023). Notoginseng leaf triterpenes ameliorates mitochondrial oxidative injury via the NAMPT-SIRT1/2/3 signaling pathways in cerebral ischemic model rats. J. Ginseng Res. 47, 199–209. 10.1016/j.jgr.2020.11.004 36926612 PMC10014186

[B57] Yamaguchi S. Irie J. Mitsuishi M. Uchino Y. Nakaya H. Takemura R. (2024). Safety and efficacy of long-term nicotinamide mononucleotide supplementation on metabolism, sleep, and nicotinamide adenine dinucleotide biosynthesis in healthy, middle-aged Japanese men. Endocr. J. 71, 153–169. 10.1507/endocrj.EJ23-0431 38191197

[B58] Yang K. Yin Q. Mao Q. Dai S. Wang L. Dong J. (2019). Metabolomics analysis reveals therapeutic effects of α-mangostin on collagen-induced arthritis in rats by down-regulating nicotinamide phosphoribosyltransferase. Inflammation 42, 741–753. 10.1007/s10753-018-09.32-2 30484004

[B59] Yao Z. Yang W. Gao Z. Jia P. (2017). Nicotinamide mononucleotide inhibits JNK activation to reverse Alzheimer disease. Neurosci. Lett. 647, 133–140. 10.1016/j.neulet.2017.0.3.027 28330719

[B60] Yi L. Maier A. B. Tao R. Lin Z. Vaidya A. Pendse S. (2023). The efficacy and safety of β-nicotinamide mononucleotide (NMN) supplementation in healthy middle-aged adults: a randomized, multicenter, double-blind, placebo-controlled, parallel-group, dose-dependent clinical trial. Geroscience 45, 29–43. 10.1007/s11357-022-00705-1 36482258 PMC9735188

[B61] Yoshino M. Yoshino J. Kayser B. D. Patti G. J. Franczyk M. P. Mills K. F. (2021). Nicotinamide mononucleotide increases muscle insulin sensitivity in prediabetic women. Science 372, 1224–1229. 10.1126/science.abe9985 33888596 PMC8550608

[B62] Zhang H. Ryu D. Wu Y. Gariani K. Wang X. Luan P. (2016). NAD⁺ repletion improves mitochondrial and stem cell function and enhances life span in mice. Science 352, 1436–1443. 10.1126/science.aaf2693 27127236

[B63] Zhang J. He Q. Mao D. Wang C. Huang L. Wang M. (2023). Efficacy and adverse reaction management of oncolytic viral intervention combined with chemotherapy in patients with liver metastasis of gastrointestinal malignancy. Front. Oncol. 13, 1159802. 10.3389/fonc.2023.1159802 37197423 PMC10183573

[B64] Zhao J. Zhang J. Yu Z. Cao Y. Chen C. Yang Z. (2018). Research and application progress of nicotinamide mononucleotides. Food Sci. Technol. 43, 257–262. 10.13684/j.cnki.spkj.2018.047

[B65] Zhao X. Zhang M. Wang J. Ji K. Wang Y. Sun X. (2022). NMN ameliorated radiation induced damage in NRF2-deficient cell and mice via regulating SIRT6 and SIRT7. Free Radic. Biol. Med. 193, 342–353. 10.1016/j.freeradbiomed.2022.10.267 36252808

[B66] Zhou B. Yang Y. Pang X. Shi J. Jiang T. Zheng X. (2023). Quercetin inhibits DNA damage responses to induce apoptosis via SIRT5/PI3K/AKT pathway in non-small cell lung cancer. Biomed. Pharmacother. 165, 115071. 10.1016/j.biopha.2023.115071 37390710

[B67] Zou X. D. Guo S. Q. Hu Z. W. Li W. L. (2016). NAMPT protects against 6-hydroxydopamine-induced neurotoxicity in PC12 cells through modulating SIRT1 activity. Mol. Med. Rep. 13, 4058–4064. 10.3892/mmr.2016.5034 27035562

